# Temporal Bone Imaging Features in Osteogenesis Imperfecta

**DOI:** 10.5334/jbr-btr.1321

**Published:** 2017-08-11

**Authors:** Isabeau Hermie, Monique Horvath, Sofie Van Cauter

**Affiliations:** 1 University Hospitals Ghent, Ghent, BE; 2 Ziekenhuizen Oost-Limburg, Genk, BE

**Keywords:** temporal bone imaging, osteogenesis imperfecta, CT, MRI, bony demineralization

## Abstract

We present the case of a 33-year old woman with osteogenesis imperfecta (OI) with progressive hearing loss and persisting vertigo. On CT-scan, symmetric extensive lucency in the pericochlear bony otic capsule and promontorium was demonstrated. The MRI-scan demonstrated symmetric areas of increased signal intensity on the T2-images with moderate contrast enhancement in the same regions. These findings correlate histologically by undermineralized thickened bone, the hallmark of OI. Hearing loss is an important clinical feature in patients with OI. The value of temporal bone imaging lies in additional confirmation of the diagnosis, determining the extent disease and excluding concomitant pathology.

## Introduction

Osteogenesis imperfecta (OI) is a rare inherited disorder of the connective tissue and is caused by an inborn error in collagen type 1 formation. Patients with OI present with a characteristic spectrum of symptoms related to deficient collagen production. Regarding vertigo and hearing loss, the related imaging features on MRI and CT of the temporal bone are typical. However, due to the rareness of the disease, these can be overlooked or wrongly considered otosclerosis.

## Case Presentation

We present the case of 33-year-old woman with genetically confirmed osteogenesis imperfecta type I. During childhood, she presented with the pathognomonic features of osteoporosis with multiple fractures and blue sclerae. At the time of consultation in our institution, she had complaints of progressive hearing loss and persist vertigo.

**Figure 1:**
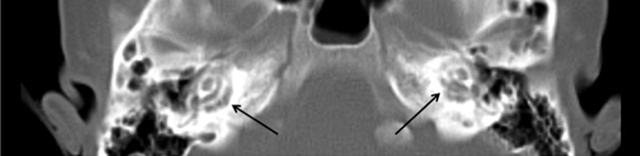
Axial CT-scan shows bilateral lucencies in the pericochlear bony otic capsule.

**Figure 2:**
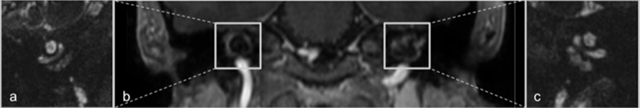
Coronal reconstructions of contrast-enhanced 3D FFE-imaging demonstrates symmetric areas of contrast enhancement in the pericochlear regions **(b)** Hyperintense signal anomalies are seen around the basal turn of the cochlea in the right **(a)** and left temporal bones **(c)** on 3D balanced steady-state gradient echo-imaging.

A spiral CT-scan with one millimetre thick sections of the temporal bone was performed. Symmetrical extensive lucencies in the pericochlear bony otic capsules, including the promontories, were demonstrated (Figure 1). An additional 3T MRI was performed and included axial FLAIR imaging, axial diffusion-weighted imaging and gadolinium-enhanced 3D fast field echo imaging (3D FFE) through the entire brain. Furthermore, 3D balanced steady-state gradient echo through the skull base completed the exam. The MRI images showed symmetric areas of increased signal intensity in the pericochlear regions on the FLAIR and 3D balanced steady-state images (Figure 2a & c). These areas showed moderate enhancement on the 3D FFE-images after contrast administration (Figure 2b).

## Discussion

Osteogenesis imperfecta (OI) is a rare inherited disorder with a prevalence of 1/10,000–30,000. It is a disorder of the connective tissue and is caused by an inborn error in collagen type 1 formation. This disorder has a very heterogeneous penetrance. There are several different collagen types, but collagen 1 (COL 1) is the most abundant and accounts for over 90% of collagen in the human body. COL 1 consists of two α–1 chains and one α–2 chain, in which each third residue is a glycine amino acid; this explains the formation of the triple helical structure. Numerous genetic mutations are identified which take part in decreased or defective type 1 collagen I formation. However, in more than 90% of the cases OI is due to an error on COL1A1 on chromosome 17 or COL1A2 gene on chromosome 7, which encodes for α–1 chains and α–2 chains respectively [1,2].

The features of the syndrome are abnormal bone fragility, osteopenia, blue sclerae, ligament laxity, dentinogenesis imperfecta and hearing impairment. The association of bone fragility, hearing loss and blue sclerae is also termed the van der Hoeve-de Kleyn syndrome [3].

Blue sclerae are the most frequent manifestation of OI. It is caused by altered light reflectance in the presence of abnormal scleral collagen [4].

OI is histopathologically characterized in general by undermineralized thickened bone. The disease alterations in the temporal bone are similar to those in the peripheral skeleton. Due to improper bone development and the presence of numerous and large vascular spaces, the bone in the mastoid region is brittle [3].

Hearing loss is an important clinical feature in patients with OI. The prevalence in several surveys has been reported between 48% to 72% [5]. Most often the condition presents as a conductive hearing loss between the second and fourth decades of life and progresses to a mixed type. However, hearing impairment in younger patients is not rare and initial presentation of mixed or sensorineural hearing loss has been reported as well [5]. Vertigo is infrequently described.

Conductive hearing loss in OI is mostly associated with footplate fixation of the stapes due to an abnormal remodelling of the temporal bone. Less frequently it results from (micro) fractures of the stapes limbs (crura), atrophy of the stapes and hypervascularized mucosa [6].

The definitive cause of sensorineural hearing loss has not been established yet, but it is thought to be a consequence of atrophy of the cochlear hair cells and the stria vascularis and abnormal bone remodelling in the cochlea and other labyrinthine structures [6].

The CT imaging of the temporal bone in OI patients with hearing loss shows reduced density in the bony regions around the semicircular canals, the distal internal auditory canal and oval window. Involvement of the fissula ante fenestram, oval window and round window (*fenestral* structures) correlates to conductive hearing loss. On the other hand, the involvement of the cochlear turns, semi-circular circles and facial canal (*retrofenestral* structures) is linked to sensorineural hearing loss [7].

The MRI imaging of the temporal bone in OI patient with hearing impairment is characterized by symmetrical, bandlike alterations in the pericochlear areas. The affected regions demonstrate T2-hyperintense signal and homogeneous contrast enhancement. The hypothesis of enhancement after contrast administration is contrast pooling in the vascular channels in the improperly mineralized bone in addition to associated inflammation [3].

The differential diagnosis of bilateral otic capsule demineralization consists of osteogenesis imperfecta, otosclerosis, Paget-disease and otosyphilis. Otosclerosis is the condition that most resembles OI, histologically as well as radiologically. However, histological analysis showed that OI involves all three bone layers of the otic capsule, while otosclerosis only involves the enchondral layer. The radiological features of the undermineralization of the otic capsule in OI and otosclerosis are similar. Nevertheless, OI is a generalized connective tissue disorder and otosclerosis is a localised disorder of the temporal bone. The extension of the involvement of the bony labyrinth is wider in OI than in otosclerosis. Clinically, the onset of hearing loss is earlier in OI than in otosclerosis [3].

## Conclusion

Patients with OI present with a characteristic spectrum of symptoms, related to deficient collagen production. Regarding vertigo and hearing loss, the related imaging features on MRI and CT of the temporal bone are typical. However, due to the rarity of the disease, these can be overlooked or considered atypical. The value of temporal bone imaging lies in additional confirmation of the diagnosis, determining the disease extent and the exclusion of concomitant pathology. Temporal bone imaging features of OI strongly resemble otosclerosis.
